# *Drosophila* Phosphatase of Regenerating Liver Is Critical for Photoreceptor Cell Polarity and Survival during Retinal Development

**DOI:** 10.3390/ijms241411501

**Published:** 2023-07-15

**Authors:** Shu-Fen Chen, Hsin-Lun Hsien, Ting-Fang Wang, Ming-Der Lin

**Affiliations:** 1 Department of Molecular Biology and Human Genetics, Tzu Chi University, 701 Zhongyang Rd., Sec. 3, Hualien 97004, Taiwan; 2013sfc@gmail.com (S.-F.C.); hhxie811@gmail.com (H.-L.H.); tingfangwang100@gmail.com (T.-F.W.); 2 Department of Life Sciences, Tzu Chi University, 701 Zhongyang Rd., Sec. 3, Hualien 97004, Taiwan; 3 Institute of Medical Sciences, Tzu Chi University, 701 Zhongyang Rd., Sec. 3, Hualien 97004, Taiwan

**Keywords:** phosphatase of regenerating liver, PRL-1, photoreceptor cell development, cell polarity

## Abstract

Establishing apicobasal polarity, involving intricate interactions among polarity regulators, is key for epithelial cell function. Though phosphatase of regenerating liver (PRL) proteins are implicated in diverse biological processes, including cancer, their developmental role remains unclear. In this study, we explore the role of *Drosophila* PRL (dPRL) in photoreceptor cell development. We reveal that dPRL, requiring a C-terminal prenylation motif, is highly enriched in the apical membrane of developing photoreceptor cells. Moreover, *dPRL* knockdown during retinal development results in adult *Drosophila* retinal degeneration, caused by *hid*-induced apoptosis. *dPRL* depletion also mislocalizes cell adhesion and polarity proteins like Armadillo, Crumbs, and DaPKC and relocates the basolateral protein, alpha subunit of Na^+^/K^+^-ATPase, to the presumed apical membrane. Importantly, this polarity disruption is not secondary to apoptosis, as suppressing *hid* expression does not rescue the polarity defect in *dPRL*-depleted photoreceptor cells. These findings underscore dPRL’s crucial role in photoreceptor cell polarity and emphasize PRL’s importance in establishing epithelial polarity and maintaining cell survival during retinal development, offering new insights into PRL’s role in normal epithelium.

## 1. Introduction

**Figure 1:**
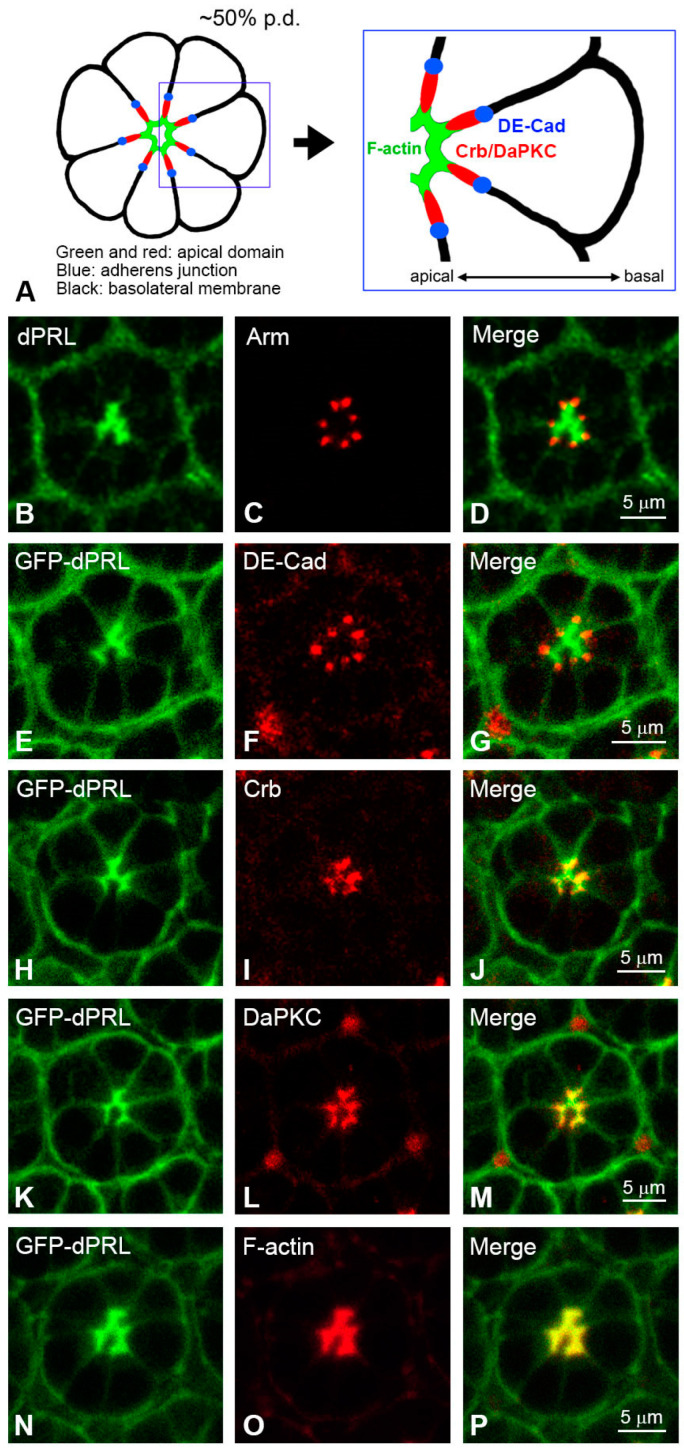
Localization of dPRL to the apical membrane of photoreceptor cells. (**A**) Schematic illustration of the cross section of a photoreceptor cluster at 50% p.d. The most apical region of the photoreceptor cells, the future rhabdomere, is enriched with F-actin (green). Localization of Crumbs (Crb)/*Drosophila* atypical protein kinase C (DaPKC) to the sub-apical membrane and that of DE-Cadherin (DE-Cad)/Armadillo (Arm) to the adherens junction (Aj) are marked with red and blue, respectively. (**B**–**P**) Analysis of colocalization between dPRL and other polarity proteins in pupal retina at 50% p.d. (**B**) The localization of endogenous dPRL was visualized by immunostaining using anti-dPRL antibody. dPRL was enriched in the apical membrane with minor localization to the basolateral membrane. Note that dPRL was also localized on the plasma membrane of pigment cells surrounding the photoreceptor cell clusters. (**E**,**H**,**K**,**N**) Localization of GFP-dPRL. Signals of dPRL came from the expression of *UAS-GFP-dPRL* driven by *GMR-Gal4* driver. (**C**,**F**,**I**,**L**,**O**) Localization of Arm, DE-Cad, Crb, and DaPKC were revealed by immunostaining; the F-actin was stained by rhodamine-conjugated phalloidin. (**D**,**G**,**J**,**M**,**P**) Merged images. Colocalization was identified in GFP-dPRL/Crb (**J**, partial colocalization), dPRL/DaPKC (**M**, partial colocalization), and dPRL/F-actin (**P**, colocalization).

*Drosophila* photoreceptor cells are specialized epithelial neurons with pronounced apicobasal polarity. A single layer of epithelial cells forms the larval eye imaginal disc, which progressively differentiates into the adult compound eye during pupal development. From 37% pupal development (p.d.) onward, the apical membranes of photoreceptor cells shift 90 degrees towards the ommatidial center and start to extend downward to the base of the ommatidium [1]. By 50% p.d., this shift is completed, and the apical membrane of the photoreceptor cells is clearly separated from the basolateral membrane by the adherens junction (AJ). The AJ coalesces to form a homogenous zonula adherens of photoreceptor epithelium. At later stages of retinal development, the apical membrane gradually subdivides into apical and sub-apical domains, which further differentiate into the photosensing rhabdomere and supporting stalk membrane of adult photoreceptor cells, respectively (Figure 1A) [1].

The establishment of apicobasal polarity in an epithelial cell relies on complex interplay among apical polarity regulators, AJ components, and basolateral polarity regulators [2]. Crumbs (Crb), an apical polarity regulator, is exclusively localized to the apical membrane and is necessary for the maintenance of the apical membrane and the formation of AJ [3,4]. *Drosophila* atypical protein kinase C (DaPKC), another apical component, is negatively regulated by its binding partner Par6 [5]. Through the interaction between Crb and Par6, DaPKC is recruited to the subapical domain of photoreceptor cells [6]. DaPKC then phosphorylates Bazooka (Baz) at serine 980 to exclude Baz from the subapical domain [6], and thus restricts Baz to AJ for assembling AJ components, such as *Drosophila* E-Cadherin (DE-Cad) and Armadillo (Arm), to form zonula adherens [7,8]. Therefore, a complex interaction among polarity regulators establishes the apicobasal polarity of the photoreceptor epithelium. However, the detailed mechanism for the specification of photoreceptor cell polarity remains to be explored.

Phosphatase of regenerating liver (PRL) proteins are part of the non-classical protein tyrosine phosphatase IVa (PTP4a) family, which functions to remove a phosphate group from its substrate protein. PRL has a shallow active site pocket, suggesting it is a dual-specific phosphatase that can act on either tyrosine or serine/threonine residues [9,10]. The mammalian PRL family consists of three homologous genes (PRL-1, -2, and -3), whereas invertebrates, like *Drosophila*, have only one PRL ortholog gene in their genome [11]. PRL family phosphatases have a conserved CX5R catalytic signature and a C-terminal CaaX motif for prenylation. Prenylation allows PRL to associate with membranous structures, such as the plasma membrane and endosomes [12,13]. Although PRL was originally identified as an immediate-early growth response gene in regenerating liver [14,15], subsequent studies have shown that the overexpression of PRL phosphatases could result in cancer metastasis by stimulating cell proliferation and migration [16,17,18].

While most PRL-related studies focus on its correlation with cancer progression, the physiological function of PRL in the developmental process remains unclear. Knockout studies in mice have shown that deficiencies of *PRL-1* [19] or *PRL-3* [20,21] do not present noticeable abnormalities during embryonic development and can survive into adulthood. By contrast, *PRL-2*-null mice exhibited defective placental development and atrophied testes with low sperm counts after birth [22,23]. In *Xenopus laevis*, although *PRL-1* and *PRL-2* have not yet been assayed, *PRL-3* is involved in controlling the migration of neural crest cells and the determination of neural crest territory [24,25]. In *Drosophila*, PRL (dPRL) is highly expressed in the developing mid-guts and central nervous system during embryogenesis [11]. In larval stage, dPRL is ubiquitously expressed in the plasma membrane of imaginal discs [11,23] and has shown apical localization in the wing imaginal disc [26]. Despite these observations, the biological significance of dPRL’s apical localization in normal epithelium has not been fully investigated.

Given the distinct polarity of *Drosophila* photoreceptor cells within the retinal epithelium, these cells present an ideal model to investigate the potential role of dPRL in the apical membrane. In this study, we demonstrate that *Drosophila* PRL exhibits an apical localization in the photoreceptor epithelium of the pupal retina at 50% pupal development (p.d.). The depletion of dPRL expression causes disruption of photoreceptor cell polarity, as evidenced by the mislocalization of polarity components. Additionally, we found that dPRL was essential for the survival of the photoreceptor epithelium, as its depletion caused a retinal degeneration phenotype. The functions of dPRL in the establishment of cell polarity and cell survival may be independent because the blockage of apoptosis could not rescue the polarity disruption phenotypes in *dPRL*-depleted photoreceptor cells. In conclusion, our study uncovered and presented a novel function of PRL in the regulation of apico-basal polarity of photoreceptor cells during *Drosophila* retinal development.

## 2. Results

### 2.1. dPRL Is Enriched in the Apical Membrane of Photoreceptor Cells at 50% Pupal Development

From 37% to 50% of pupal development (p.d.), the apical membrane of the photoreceptor cell undergoes a 90-degree shift towards the ommatidial center [1]. This membrane is enriched with F-actin and can be easily demarcated by the adherens junctions (AJs) marked by *Drosophila* β-catenin Armadillo (Arm) or *Drosophila* E-Cadherin (DE-Cad). The AJs delineate the boundary between the apical and basolateral membranes. Following 50% p.d., the apical membrane of photoreceptor cells further differentiates into the rhabdomere, enriched with F-actin, and the subapical stalk membrane, marked by Crumbs (Crb) or *Drosophila* atypical protein kinase C (DaPKC) [1] (Figure 1A).

At 50% p.d., endogenous dPRL is predominantly localized in the apical membrane at the ommatidia center, with residual localization in the basolateral membrane (Figure 1B). This localization is adjacent to the adherens junctions, as marked by Arm (Figure 1C,D). To further validate dPRL’s apical localization, we examined the localization of GFP-tagged dPRL in the retina, using the pan-retinal driver *GMR-Gal4* [27] to drive the expression of the *UAS-GFP-dPRL* transgene. Consistent with the localization of endogenous dPRL at 50% p.d., the majority of the GFP-dPRL signal (Figure 1E) was concentrated in the apical membrane, outlined by the presence of the adherens junction component, DE-Cad (Figure 1F,G). Within the apical membrane, GFP-dPRL (Figure 1H,K) partially co-localized with the subapical stalk membrane markers Crb (Figure 1I,J) and DaPKC (Figure 1L,M). Furthermore, GFP-dPRL (Figure 1O,P) was found to co-localize with F-actin in the apical membrane.

### 2.2. The Prenylation Motif Is Essential for dPRL’s Localization to the Apical Membrane of Photoreceptor Cells

**Figure 2:**
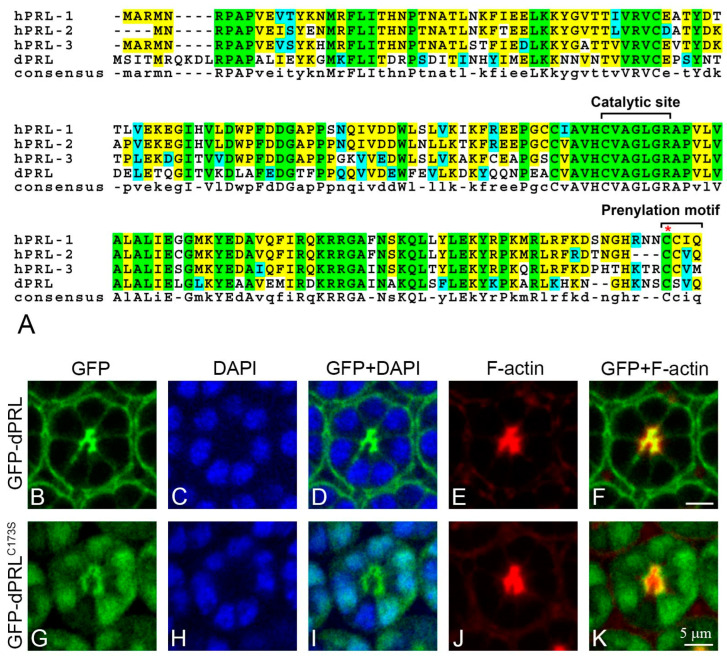
The subcellular localization of GFP-dPRL and GFP-dPRL^C173S^ in photoreceptor cells. (**A**) Multiple sequence alignment of PRL proteins in humans and *Drosophila*. The C(X)5R phosphatase catalytic site and the CAAX prenylation motif are highlighted. The position of Cys173 in dPRL is marked with a red asterisk. Green represents identical residues, blue indicates residues with similar properties, and yellow marks consensus residues. (**B**–**K**) Immunostaining was performed on the pupal retina at 50% p.d. GFP is depicted in green, DAPI in blue, and F-actin in red. (**B**–**F**) Pupal retina expressing GFP-tagged wild-type dPRL, genotype: *GMR-Gal4/+; UAS-GFP-dPRL/+*. (**B**) GFP-dPRL was localized to the plasma membrane, with marked enrichment at the apical membrane of photoreceptor cells. (**C**) Nuclei were stained with DAPI. (**D**) Merged image of (**B**,**C**). (**E**) F-actin was highly enriched at the apical membrane of photoreceptor cells. (**F**) Merged image of (**B**,**E**), indicating co-localization of dPRL with F-actin at the apical membrane. (**G**–**K**) Pupal retina expressing GFP-tagged dPRL carrying a Cys173 to Ser substitution, genotype: *GMR-Gal4/+; UAS-GFP-dPRL^C173S^/+*. (**G**) GFP-dPRL^C173S^ was found to be mislocalized in the photoreceptor cells, notably accumulating in the cytosol near the apical membrane and within the nucleus. (**H**) Nuclei were stained with DAPI. (**I**) Merged image of (**G**,**H**). (**J**) F-actin staining. (**K**) Merged image of (**G**,**J**), demonstrating that GFP-dPRL^C173S^ did not co-localize with F-actin. Scale bars represent 5 µm.

The C-terminal CAAX prenylation motifs in mammalian PRLs are known to be required for their association with membranous structures (Figure 2A) [12,13,28,29]. To investigate whether the C-terminal CSVQ sequence of dPRL is required for its association with the apical membrane in photoreceptor cells, we generated *UAS-GFP-dPRL^C173S^* transgenic flies carrying a Cys173 to Ser substitution to disrupt the prenylation motif. In photoreceptor cells of the developing retinas at 50% p.d., GFP-dPRL was primarily localized to the apical membrane, co-localizing with F-actin (Figure 2B–F). In contrast, GFP-dPRL^C173S^ accumulated in the cytosol adjacent to the apical membrane (Figure 2G,J,K) and was even mislocalized to the nucleus (Figure 2G–I). These results indicate that the C-terminal CSVQ sequence is crucial for targeting dPRL to the apical membrane of photoreceptor cells during retinal development.

### 2.3. Knockdown of dPRL Leads to Retinal Degeneration

**Figure 3:**
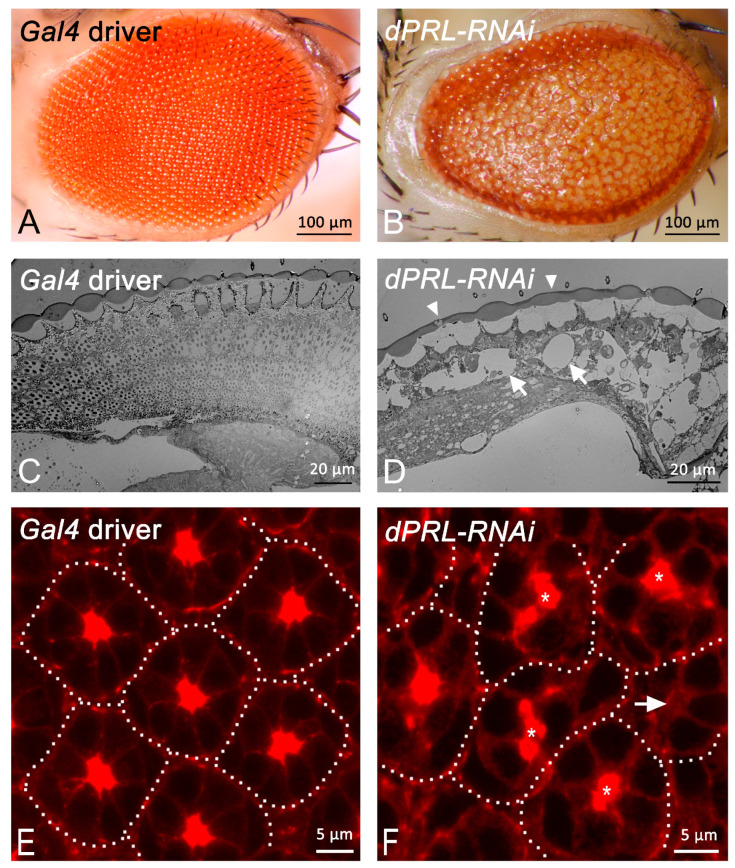
Retinal phenotypes of *dPRL-RNAi* flies. (**A**,**B**) Comparison of eye phenotypes between the driver control and *dPRL-RNAi* flies. (**A**) *Gal4*-driver control (*UAS-dicer2; GMR-Gal4/+*), demonstrating a wild-type phenotype. (**B**) *dPRL-RNAi* flies (*UAS-dicer2; GMR-Gal4/+; UAS-dPRL-IR^45518^/+*), showing loss of pigmentation, thinning, and roughened eyes. (**C**,**D**) Interior structures of adult retinas, as seen in longitudinal sections imaged using a transmission electron microscope. (**C**) *Gal4*-driver control (*UAS-dicer2; GMR-Gal4/+*). (**D**) Retina of *dPRL-RNAi* flies, exhibiting vacuoles (indicated by arrows) and a fused cornea (indicated by arrowheads). (**E**,**F**) Pupal retinas at 50% p.d (pupal development) with F-actin (red) localization visualized by rhodamine-conjugated phalloidin staining. Dotted lines outline individual ommatidia. (**E**) Wild-type pupal retina exhibits a hexagonal array of ommatidia with F-actin concentrated at the apical membrane of photoreceptor cells. (**F**) In the pupal retina of *dPRL-RNAi* flies, ommatidia are irregularly arranged and F-actin is either mislocalized (asterisk) or absent (arrow).

To elucidate the potential role of dPRL in retinal development, we employed an in vivo RNA interference (RNAi) strategy to suppress *dPRL* expression in the retina. The successful knockdown of *dPRL* was confirmed through immunostaining, as demonstrated in Figure S1E. In these experiments, we utilized *GMR-Gal4* to drive the simultaneous expression of *UAS-dPRL-IR^45518^* and *UAS-dicer2* transgenes, generating *dPRL* inverted-repeat (IR) RNA sequences for *dPRL* knockdown and Dicer 2 for enhancing RNAi efficiency, respectively [30]. For simplicity, this specific genetic combination is referred to as *dPRL-RNAi*. In the *GMR-Gal4; UAS-dicer2* control flies, no discernible abnormalities in the compound eyes were observed (Figure 3A). Conversely, *dPRL-RNAi* flies exhibited noticeable compound eye aberrations, including eye roughening, thinning, and loss of pigmentation (Figure 3B). These phenotypes were successfully reproduced using a distinct RNAi line, *UAS-dPRL-IR^107836^*, which carries an IR sequence of *dPRL* that differs from that of *UAS-dPRL-IR^45518^*.

To gain a deeper understanding of the eye aberration phenotypes observed in *dPRL-RNAi* flies, we employed transmission electron microscopy for examination. The adult retina of *Gal4*-driver control flies displayed a regular array of ommatidia (Figure 3C). Contrarily, the adult retina of *dPRL-RNAi* flies was characterized by the presence of numerous vacuoles (Figure 3D, arrows), indicative of severe degeneration, and deterioration of the cornea and pseudocone was also noted (Figure 3D, arrowheads). In addition to the adult eye, we examined the pupal retina of *dPRL-RNAi* flies. The pupal retina of *GMR-Gal4; UAS-dicer2* control flies at 50% p.d. exhibited a regular hexagonal array of ommatidia (Figure 3E), and the F-actin, which is enriched in the apical membrane of the photoreceptor cell, was readily observable at the center of each ommatidium (Figure 3E, arrows). However, in the pupal retina of *dPRL-RNAi* flies at 50% p.d., the ommatidia were irregularly positioned (Figure 3F) and the F-actin was mislocalized (Figure 3F, arrows). Collectively, our results demonstrate that the knockdown of *dPRL* in the developing retina disrupts the regular array of ommatidia in the pupal retina and induces retinal degeneration in adult compound eyes.

### 2.4. Knockdown of dPRL Triggers Apoptosis Mediated by the Proapoptotic Gene Hid

**Figure 4:**
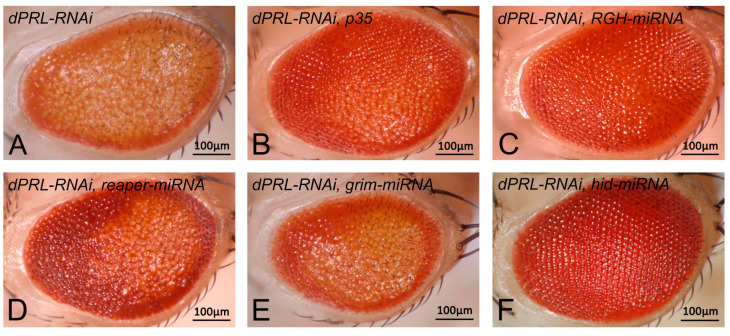
Knockdown of the proapoptotic gene *hid* rescued the loss-of-pigmentation phenotype observed in *dPRL-RNAi* flies. (**A**) Eye phenotype of *dPRL-RNAi* flies. (**B**–**F**) Effects of expressing the anti-apoptotic protein *p35* or knocking down proapoptotic genes using miRNAs in *dPRL-RNAi* flies. (**B**) Eye phenotype following ectopic expression of the anti-apoptotic gene *p35*. (**C**) Eye phenotype after combined knockdown of proapoptotic genes *reaper*, *grim*, and *hid* using a miRNA construct (*RGH-miRNA*). (**D**) Eye phenotype following *reaper* knockdown using miRNA. (**E**) Eye phenotype following *grim* knockdown using miRNA. (**F**) Eye phenotype after *hid* knockdown using miRNA. Eye phenotypes were successfully rescued in panels (**B**,**C**,**F**). There is a high degree of phenotypic consistency (>80%) within each genotype. The images displayed herein represent the typical eye morphology for each respective genotype.

The retinal degeneration phenotype observed in *dPRL-RNAi* flies suggests that apoptosis could be the cause. To validate this, we used the baculovirus anti-apoptotic protein p35 to potentially reverse the eye phenotype of *dPRL-RNAi* flies. We observed that the expression of p35 mitigated the loss-of-pigmentation phenotype in *dPRL-RNAi* flies (Figure 4A,B), indicating that *dPRL*-knockdown-induced retinal degeneration primarily results from apoptosis. In *Drosophila*, apoptosis can be triggered by the expression of proapoptotic genes such as *reaper*, *grim*, and *hid*. To determine whether these proapoptotic genes were activated following *dPRL* knockdown, we used a microRNA technique [31] to suppress the expression of these genes and assess the impact on the eye degeneration phenotype of *dPRL-RNAi* flies. The result showed that the concurrent knockdown of *reaper*, *grim*, and *hid* fully reversed the loss-of-pigmentation phenotype of *dPRL-RNAi* flies (Figure 4C). However, the individual knockdown of either *reaper* (Figure 4D) or *grim* (Figure 4E) was insufficient to completely rescue the eye defects of *dPRL-RNAi* flies. Interestingly, the sole knockdown of *hid* significantly mitigates the eye phenotype of *dPRL-RNAi* flies (Figure 4F). Our results suggest that the proapoptotic gene *hid* is involved in the retinal degeneration observed following *dPRL* knockdown.

### 2.5. Polarity of Photoreceptor Cells was Disrupted in the Pupal Retina of dPRL-Knockdowned Flies

**Figure 5:**
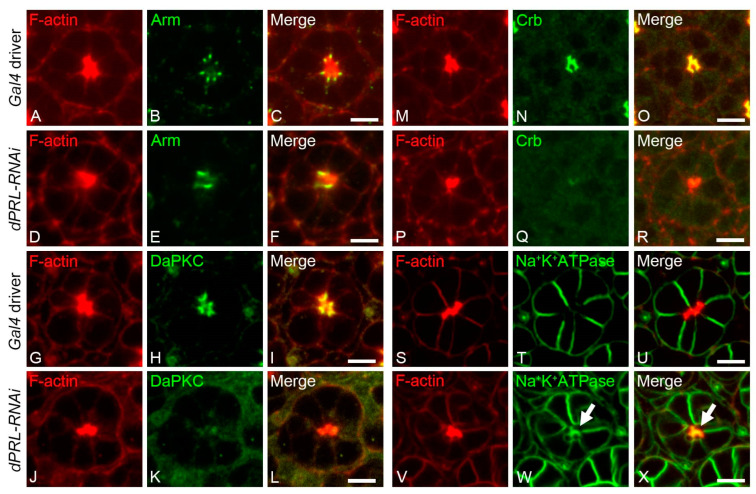
Mislocalization of apicobasal polarity proteins in the photoreceptor cells of *dPRL-RNAi* flies. Immunostaining was conducted on pupal retinas from either the *Gal4*-driver control (*UAS-dicer2; GMR-Gal4/+*) or *dPRL-RNAi* flies (*UAS-dicer2; GMR-Gal4/+; UAS-dPRL-IR^45518^/+*) to illustrate the localization of polarity proteins in photoreceptor cell clusters at 50% p.d. (**A**–**F**) Pupal retinas from *Gal4*-driver control (**A**–**C**) and *dPRL-RNAi* flies (**D**–**F**) were stained with phalloidin for labelling F-actin (**A**,**C**,**D**,**F**; red) and with an antibody against Arm (**B**,**C**,**E**,**F**; green). (**G**–**L**) Pupal retinas from *Gal4*-driver control **(G**-**I**) and *dPRL-RNAi* flies (**J**-**L**) were stained with phalloidin for labelling F-actin (**G**,**I**,**J**,**L**; red,) and an antibody against DaPKC (**H**,**I**,**K**,**L**; green). (**M**–**R**) Pupal retinas from *Gal4*-driver control (**M**–**O**) and *dPRL-RNAi* flies (**P**–**R**) were stained with phalloidin for labelling F-actin (**M**,**O**,**P**,**R**; red) and an antibody against Crb (**N**,**O**,**Q**,**R**; green). (**S**–**X**) Pupal retinas from *Gal4*-driver control (**S**–**U**) and *dPRL-RNAi* flies (**V**–**X**) were stained with phalloidin for labelling F-actin (**S**,**U**,**V**,**X**; red) and an antibody against the alpha subunit of Na^+^/K^+^-ATPase (**T**,**U**,**W**,**X**; green). Arrows in panels W and X highlight the mislocalization of the Na^+^/K^+^-ATPase (green) to the apical membrane, which is marked by F-actin (red). Scale bars represent 5 µm.

Given the predominant localization of dPRL in the apical membrane of developing photoreceptor cells, we hypothesized its potential role in establishing photoreceptor polarity. We first inspected the AJ components in the pupal retina of *dPRL-RNAi* flies at 50% p.d. Compared to the *GMR-Gal4; UAS-dicer2* control retina (Figure 5A–C), we observed significant mislocalization of the AJ component, Arm, in the *dPRL-RNAi* retina (Figure 5E). In some ommatidia of *dPRL-RNAi* flies, the AJ between adjacent photoreceptor cells was either fused or could not be identified (Figure 5F). Further, we examined the sub-apical membrane integrity of photoreceptor cells in the ommatidia of *dPRL-RNAi* flies and found that both DaPKC and Crb lost their sub-apical membrane localization (DaPKC, Figure 5J–L; Crb, Figure 5P–R) compared to the control retinas (DaPKC, Figure 5G–I; Crb, Figure 5M–O). The disruption of AJs, which delineate the boundary between the apical and basolateral membranes, could imply the mislocalization of basolateral membrane proteins to the apical membrane. In the *GMR-Gal4; UAS-dicer2* control retina, the alpha subunit of the Na^+^/K^+^-ATPase was specifically localized to the basolateral membrane [32] (Figure 5S–U). However, in *dPRL-RNAi* flies, we observed the mislocalization of the alpha subunit of the Na^+^/K^+^-ATPase to the apical membrane, where it colocalized with F-actin in the photoreceptor cells (Figure 5V–X, arrow). These results suggest that dPRL is vital for the apicobasal polarity of photoreceptor cells, and its depletion could disrupt AJs, leading to the mislocalization of polarity-determining proteins.

### 2.6. Polarity Disruption of Photoreceptor Cells in dPRL-Depleted Retina was Not a Secondary Effect of Apoptosis

**Figure 6:**
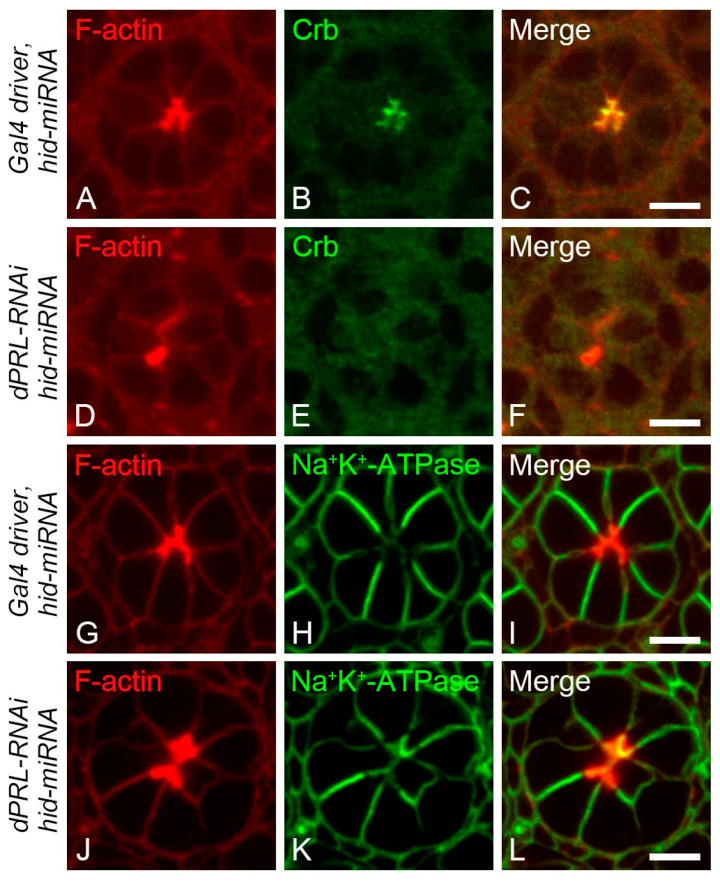
Suppression of apoptosis could not rescue the photoreceptor cell polarity defect caused by *dPRL* knockdown. (**A**–**C**,**G**–**I**) Show representative ommatidia from control flies expressing the *Gal4* driver and *hid-miRNA* (genotype: *UAS-dicer2*; *GMR-Gal4/+*; *UAS-hid-miRNA/+*). (**D**–**F**,**J**–**L**) Show representative ommatidia from *dPRL-RNAi* flies expressing *hid-miRNA* (genotype: *UAS-dicer2*; *GMR-Gal4/+*; *UAS-hid-miRNA/UAS-dPRL-IR^45518^*). Pupal retinas at 50% p.d. were fixed and stained for F-actin with rhodamine-conjugated phalloidin to mark the apical membrane, along with Crumbs (Crb, green) in (**A**–**F**), or the alpha subunit of Na^+^/K^+^-ATPase (green) in (**G**–**L**). Notably, despite suppression of apoptosis through the expression of *hid-miRNA*, Crb (**E**) and Na^+^/K^+^-ATPase (**K**) were still mislocalized in the *dPRL*-knockdown retina. Scale bars represent 5 µm.

Given that the silencing of dPRL disrupted both photoreceptor cell polarity and induced apoptosis, we questioned whether the polarity disruption could be a secondary effect resulting from cell death. To test this hypothesis, we suppressed apoptosis in *dPRL*-knockdown flies by inhibiting *hid* expression, as shown in Figure 4F, then assessed photoreceptor cell polarity in pupal retinas. In control pupal retinas at 50% p.d. from flies carrying the *GMR-Gal4* driver with *UAS-dicer2* and *UAS-hid-miRNA* transgenes, the polarity of photoreceptor cells was normal, with both the sub-apical marker Crb (Figure 6A–C) and the basolateral membrane marker, the alpha subunit of Na^+^/K^+^-ATPase (Figure 6G–I), localizing properly. However, in photoreceptor cells of *dPRL-RNAi* flies carrying a *UAS-hid-miRNA* transgene, we observed a reduction in Crb expression in the sub-apical membrane (Figure 6D–F) and a mislocalization of the alpha subunit of Na^+^/K^+^-ATPase (Figure 6J–L) to the apical membrane. These results suggest that the disruption in photoreceptor cell polarity caused by *dPRL* knockdown operates independently of apoptosis.

## 3. Discussion

In this study, we demonstrate that dPRL is primarily localized to the apical membrane of developing photoreceptor cells. Regarding dPRL’s role in the development of the retina, we found that its depletion disrupts the apico-basal polarity of photoreceptor cells. PRL’s function in the establishment of apical-basal polarity in the normal epithelium could potentially be universal, as evidenced by the formation of multiple ectopic apical membrane initiation sites enriched with PRL-3 when human *PRL-3* is overexpressed in 3D-cultured non-cancerous Madin–Darby canine kidney (MDCK) cells [33]. Given that the disruption of epithelial architecture is a fundamental event in epithelial tumorigenesis, our findings establish a connection between PRL’s normal function in epithelial cells and its role in human cancers.

The establishment of epithelial cell polarity is known to be contingent on a complex interplay between components of cell adhesion and cell polarity complexes. DaPKC, one of these components, plays a pivotal role in forming adherens junctions (AJs) during the remodeling of apico-basal polarity in photoreceptor cells. Specifically, DaPKC phosphorylates Baz, excluding it from the apical membrane, which in turn confines Baz to the future AJ through Crb activity [6]. Following this, Baz recruits components such as Arm and DE-Cad to establish the AJ [7,8]. In pupal eye discs with *dPRL* depletion, we observed the disruptions of AJs (Figure 4D–F), accompanied by a loss of apical localization of both DaPKC and Crb in photoreceptor cells (Figure 4J–L,P–R). The disruption of photoreceptor cell polarity of *dPRL-RNAi* flies can be traced back to at least 40% p.d., a time point that immediately follows the onset of the apical membrane shift observed at 37% p.d. (Figure S1). These findings suggest that dPRL acts genetically upstream of DaPKC and Crb in the establishment of photoreceptor cell polarity. The observed disruption of AJs in *dPRL*-depleted photoreceptor cells could potentially be explained by the absence of DaPKC from the apical membrane. GSK3β, known to directly phosphorylate DaPKC, triggers its ubiquitin-mediated proteasomal degradation. This process is pivotal in the establishment of apical-basal polarity during *Drosophila* embryogenesis [34,35]. Interestingly, AKT has been found to phosphorylate and inhibit GSK3β [36]. It is conceivable that PRL phosphatases could modulate this pathway. Supporting this notion, studies have found that PRL-2 in mouse placenta [23] and human PRL-3 in HeLa cells [37] can activate AKT kinase through the downregulation of PTEN, a well-established antagonist of the PI3K pathway. Furthermore, human PRL-3 has been reported to interact with and deactivate the SHP2 phosphatase, a negative regulator of EGF-dependent PI3K activation [38,39,40]. Based on these studies, we postulate that dPRL may influence the AKT-GSK3β-DaPKC pathway either directly or indirectly. This, in turn, could alter the localization and stability of DaPKC at the apical membrane, impacting apical-basal polarity. Alternatively, dPRL might influence AJ formation through interactions with Cadherin, a notion supported by studies showing that human PRL-3 has been implicated in the epithelial–mesenchymal transition (EMT) via a Cadherin-related signaling pathway [37,41]. However, the exact mechanisms by which dPRL influences DaPKC localization, and whether they directly interact, remain to be determined.

We also found that *dPRL* depletion induced cell death in the developing retina (Figure 3B,D). This effect could be mitigated by either overexpressing the caspase inhibitor *p35* or by silencing the proapoptotic gene *hid* via miRNA (Figure 4), suggesting that *dPRL* depletion triggers *hid*-mediated apoptosis. Several pathways may be responsible for the induction of apoptosis following *dPRL* depletion, including the c-Jun N-terminal kinase (JNK) signaling pathway and p53, both of which have previously been reported to induce *hid* expression. JNK signaling has been shown to promote *hid* transcription following UV exposure in the developing retina [41], while p53 has been reported to induce *hid* expression in embryos or larvae after radiation exposure [42,43]. This idea is further supported by a previous study demonstrating that mouse *PRL-3* knockdown triggers cell cycle arrest through the upregulation of *p53* [44]. Additionally, the apoptosis-mediated retinal degeneration observed in *dPRL*-depleted retinas could be due to direct or indirect disruptions to photoreceptor cell polarity. This possibility is supported by findings showing that cells with polarity deficiencies trigger apoptosis via JNK signaling activation [45,46,47]. Considering the hypothesis that disruptions in epithelial polarity might trigger apoptosis to remove potential tumor cells, our study offers insights into the developmental functions of PRL in both normal epithelia and cancer progression. Further research is needed to fully elucidate how dPRL regulates cell polarity and the intimate relationship it has with cell survival.

## 4. Materials and Methods

### 4.1. Drosophila Stocks

*Drosophila* stocks were maintained on a standard cornmeal medium. The following transgenic stocks were utilized in this study: *P{w+; UAS-GFP::dPRL^WT^}*, *P{w+; UAS-GFP::dPRL^C173S^}*, *P{w+; GMR-RGH-miRNA}* [34], *P{w+; GMR-reaper-miRNA}* [34], *P{w+; GMR-grim-miRNA}* [34], *P{w+; GMR-hid-miRNA}* [34], *P{w+; UAS-dPRL-IR^45518^}* (Vienna *Drosophila* Resource Center (VDRC, Vienna, Austria, stock No. 45518), *GMR-Gal4* (Bloomington *Drosophila* Stock Center (BDSC, Bloomington, IN, USA, stock No. 1104), *UAS-dicer2* (BDSC, stock No. 24646), *P{w+; UAS-p35}* (BDSC, stock No. 5072). To ensure the *GAL4* expression and efficiency for RNA interference, flies were raised at 27 °C for pupal dissection. The wild-type coding sequence of *dPRL* were subcloned into the *pUAST* vector containing a *GFP* coding sequence upstream of the cloning site for transgenes. The QuikChange Lightning Site-Directed Mutagenesis kit (Agilent Technologies, Santa Clara, CA, USA) was used to generate the Cys173 to Ser substitution in the *dPRL* coding sequence. Transgenic flies were generated according to the standard protocol via microinjection.

### 4.2. Transmission Electron Microscopy (TEM) for Adult Retina

Adult flies were fixed in a solution containing 2% paraformaldehyde and 2% glutaraldehyde in 0.1 M cacodylate buffer, pH 7.3, and incubated at 4 °C. Subsequent to hand dissection, the heads were subjected to an additional 2 h incubation in fixative at 4 °C. This was followed by post-fixation with 1% OsO4 in 0.1 M cacodylate buffer at room temperature. The heads were then stained en bloc with 2% aqueous uranyl acetate for 1 h, followed by a series of dehydration steps using alcohol, and finally embedded in Spurr’s resin (Electron Microscopy Sciences, Hatfield, PA, USA, Cat. No. 14300). Eye sections were prepared using a Leica EM UC6 ultramicrotome and visualized using a Hitachi H7500 transmission electron microscope.

### 4.3. Drosophila Whole-Mount Immunostaining of Pupal Retina

Pupal retinas at the specified developmental stages were hand-dissected and fixed for 20 min in 4% formaldehyde. Upon fixative removal, the fixed retinas were washed multiple times in PBST (PBS containing 0.3% Triton X-100). Retinas were then blocked with 2% bovine serum albumin in PBST for 1 h and incubated overnight at 4 °C with primary antibodies diluted in PBS. After incubation, the retinas were washed three times for 20 min each in PBST and subsequently incubated for 2 h at room temperature with secondary antibodies in PBST. Following three additional 30 min washes in PBST, retinas were mounted using an anti-fade mounting solution. The primary antibodies used were rabbit anti-dPRL (1:100 dilution) [11], mouse anti-Arm (Developmental Studies Hybridoma Bank (DSHB), Iowa, IA, USA, Product ID: N27-A1, 1:50 dilution), mouse anti-alpha subunit of Na^+^/K^+^-ATPase (DSHB, Product ID: a5, 1:20 dilution), rat anti-DE-Cad (DSHB, Product ID: DCAD2, 1:20 dilution), mouse anti-Crumbs (Developmental Studies Hybridoma Bank, Product ID: Cq4, 1:20 dilution), and rabbit anti-DaPKC (Santa Cruz, Dallas, TX, USA, Cat. No. SC-216, 1:50 dilution). The fluorescent-labeled secondary antibodies used were goat-anti-rabbit Alexa Fluor 488 (Invitrogen, Waltham, MA, USA, Cat. No. A-11008, 1:400 dilution), goat anti-rat Alexa Fluor 633 (Invitrogen, Cat. No. A-21094, 1:400 dilution), and goat anti-mouse Alexa Fluor 633 (Invitrogen, Cat. No. A-21050, 1:400 dilution). For nuclear stain, DAPI solution (1 mg/mL) was used (Sigma-Aldrich, Burlington, MA, USA, Cat. No. MBD0015, 1:1000 dilution). F-actin was labeled with rhodamine-conjugated phalloidin (Sigma-Aldrich, Cat. No. 77418, 1:200 dilution).

## Data Availability

Data are contained within the article.

## Supplementary Materials

The following supporting information can be downloaded at: https://www.mdpi.com/article/10.3390/ijms241411501/s1.
