# Supplementary material for: Drosophila Phosphatase of Regenerating Liver Is Critical for Photoreceptor Cell Polarity and Survival during Retinal Development
Source: Int J Mol Sci. 2023 Jul 15;24(14):11501. doi: 10.3390/ijms241411501 (PMC10380645; doi:10.3390/ijms241411501)
Supplement: Supplementary file 1 [file ijms-24-11501-s001.zip › ijms-2494791-supplementary.pdf]

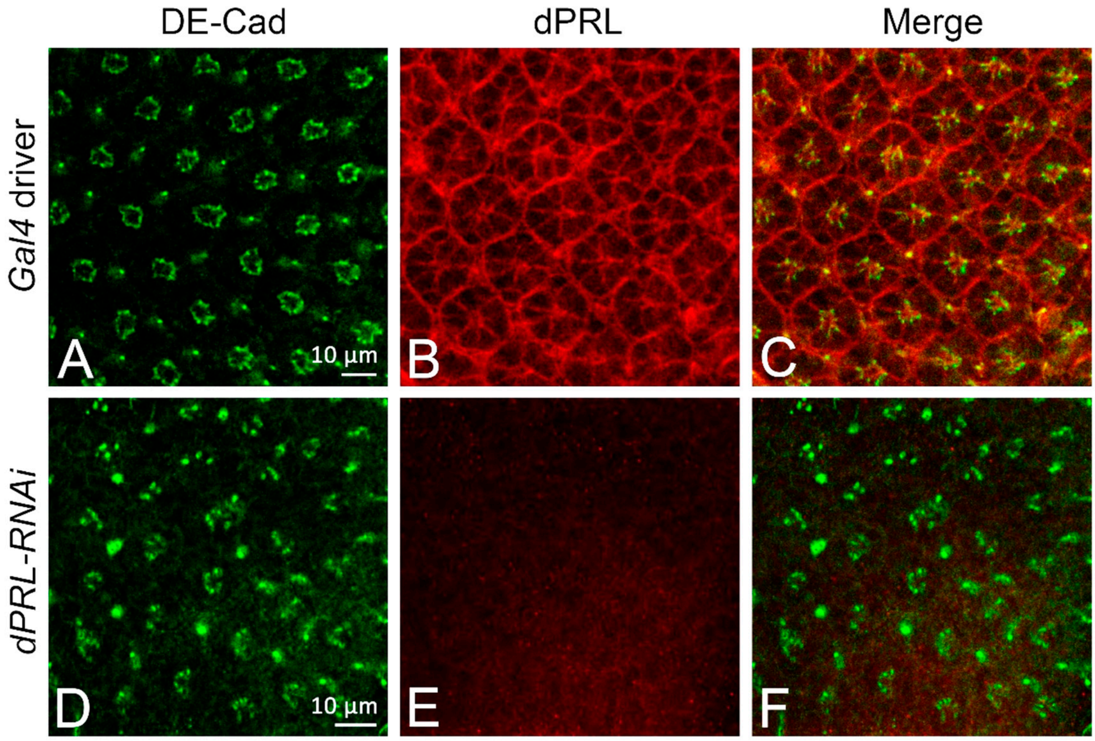

Figure S1. Depletion of *dPRL* results in mislocalization of *Drosophila* E-Cadherin in photoreceptor clusters at 40% pupal development (p.d.). Immunostaining was conducted on pupal retinas obtained from either the *Gal4*-driver control (*UAS-dicer2*; *GMR-Gal4*/+) or *dPRL-RNAi* (*UAS-dicer2*; *GMR-Gal4*/+; *UAS-dPRL-IR*^{45518}/+) flies to demonstrate the localization of *Drosophila* E-Cadherin (DE-Cad; green) and dPRL (red) in photoreceptor cell clusters at 40% p.d. Pupal retinas from *Gal4*-driver control (A-C) and *dPRL-RNAi* (D-F) flies were stained with anti-DE-Cad (A and D; green) and anti-dPRL (B and E; red) antibodies. (C and F) represent merged images. The mislocalization of DE-Cad (D) and the reduction of dPRL (E) were evident in the pupal retina at 40% p.d. Scale bars represent 10 µm.
